# Hydrogen Atom Abstraction Reaction from Silane with Hydrogen and Methyl Radicals: Rate Constants and Kinetic Isotopic Effects

**DOI:** 10.1021/acs.jpca.4c05382

**Published:** 2024-11-29

**Authors:** Filipe G. Kano, Edson F. V. de Carvalho, Luiz F. A. Ferrão, Orlando Roberto-Neto, Francisco B. C. Machado

**Affiliations:** † Departamento de Química, Instituto Tecnológico da Aeronáutica, São José dos Campos, São Paulo 12228-900, Brasil; ‡ Laboratório de Computação Científica Avançada e Modelamento (Lab-CCAM), Instituto Tecnológico da Aeronáutica, São José dos Campos, São Paulo 12228-900, Brasil; § Departamento de Física, Universidade Federal do Maranhão, São Luís, Maranhão 65085-580, Brasil; ∥ Divisão de Aerotermodinâmica e Hipersônica, Instituto de Estudos Avançados, São José dos Campos, São Paulo 12228-001, Brasil

## Abstract

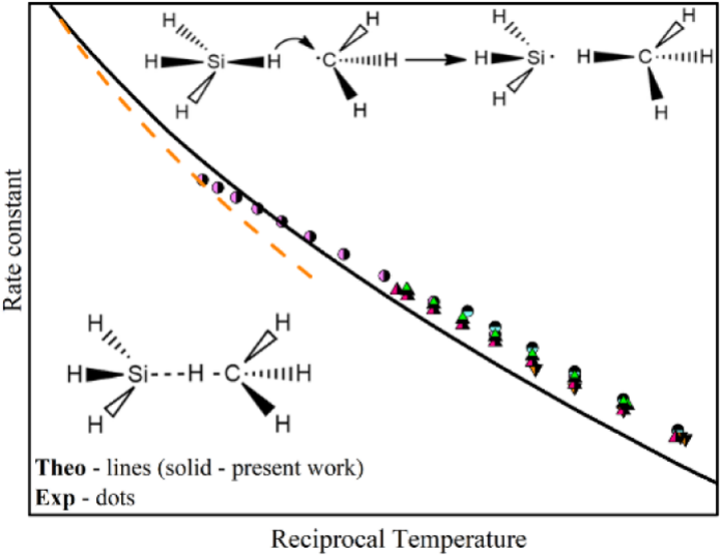

The thermal rate constants and H/D kinetic isotope effects (KIEs) of the reactions SiH_4_ + H  SiH_3_ + H_2_ and SiH_4_ + CH_3_ SiH_3_ + CH_4_ were calculated with the variational transition state theory including multidimensional tunneling corrections (VTST-MT). The ωB97X-D and CCSD(T) methods were employed to compute the barrier height and reaction energy. The ωB97X-D/aug-cc-pVTZ methodology was used to build reaction paths, and the CCSD(T)/CBS_Q-5_ approach was used to improve the energetics of the stationary points in the calculations of CVT/μOMT thermal rate constants. For the SiH_4_ + H pathway, the CVT/μOMT rate constants and H/D KIEs are in excellent agreement with previous experimental and theoretical calculations; otherwise, for the SiH_4_ + CH_3_ pathway, which has few experimental and theoretical data available, it has provided, for the first time, rate constants and kinetic isotope effects using variational transition state theory with multidimensional tunneling corrections.

## Introduction

Silane (SiH_4_) is a colorless flammable gas classified as dangerous by the European GHS classification and labeling. However, according to Global Market Insights (GMI), the market for silane in 2020 was approximately 2.37 billion USD and will exhibit a compound annual growth rate of around 7.8% between 2021 and 2027.^1^

Typically used as a precursor compound for the synthesis of silicon-containing nanoparticles and coatings,^2^ silane has several applications in the semiconductor industry for dielectric layers. In the aerospace sector, silane has been proposed as an ignition source^3^ and fuel igniter for scramjet engines.^4^ Because of its wide range of applications, the elucidation of oxidation mechanisms is highly relevant. Experimental and theoretical investigations, especially those involving elementary abstraction reactions with silane, have been the focus of numerous significant research efforts in recent decades.^2−17^

The elementary reaction SiH_4_ + H in the gas phase is an important step in the mechanism of thermal decomposition of monosilane and plays a significant role in the chemical vapor deposition (CVD) reactive mechanism widely used in semiconductor industries.^6−8^ Its kinetics has been used as a reaction model in the experimental^2,9,13^ and benchmark theoretical studies of combustion and reactive processes involving silane.^5,11,12^

Experimentally, Goumri et al.,^9^ employing flash photolysis-resonance fluorescence techniques, studied its kinetics in the temperature range of 290–660 K and obtained  x 10^–13^ cm^3^molecule^–1^s^–1^ as the rate constant at a temperature of 290 K. Arthur et al.,^13^ using a similar technique, measured the rate constants in the same temperature range of 298 to 636 K, obtaining a value of  x 10^–13^ cm^3^molecule^–1^s^–1^ at room temperature. Peukert et al.,^2^ using a shock-tube technique, investigated this reaction at high temperatures, in the range of 1170 to 1251 K.

Theoretically, for the reactions from (R1) to (R1c), Espinosa-García et al.^5^ employed variational transition state theory (VTST) with a full-dimensional analytical potential energy surface (PES) to compute the rate constants, and they obtained a global rate constant equal to 2.09 × 10^–13^ cm^3^ mol^–1^s^–1^ at 300 K. Using the same PES, Wang et al.^11^ employed the quantum instanton (QI) method to calculate the rate constants and obtained a global rate constant value equal to 2.74 × 10^–13^ cm^3^ mol^–1^s^–1^ at 300 K. Cao et al.,^12^ with a global 12-dimensional *abinitio* PES, employed VTST for the calculations of rate constants and obtained a global rate constant value equal to 1.72 × 10^–13^ cm^3^ mol^–1^s^–1^ at 300 K.
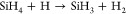
R1
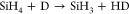
R1a
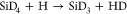
R1b
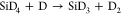
R1cOn the other hand, quantitative kinetic data for SiH_4_ + CH_3_ are sparse. Recently, in a study conducted by Fang et al.,^18^ a theoretical investigation was carried out, which highlighted the selection of the H and CH_3_ radicals as the two attacking radicals to reveal the influence of different radicals on the overall H abstraction reactions of silanes and their alkane counterparts. The analysis revealed that the energy obtained at the DLPNO–CCSD(T)/cc-pVTZ//M06-2X/cc-pVTZ level for these H abstraction reactions from primary carbon sites in silanes generally exhibits barrier energies lower than those of similar reactions in their alkane counterparts. The rate constants adjusted for the silane H abstraction reactions were obtained using conventional transition-state theory coupled with Eckart asymmetric tunneling corrections from 600 to 2000 K. For the reactions SiH_4_ + H and SiH_4_ + CH_3_, the Arrhenius fit parameters of the rate constants in cm^3^molecule^–1^s^–1^ are, respectively, *k*(*T*) =  and 

Experimentally,^10,14−17^ the rate constants have been evaluated indirectly by the competitive method in relation to the rate constants of the radical recombination of the 2 C_2_H_6_ reaction.^10^ Furthermore, the study of this reaction (R2) is essential to better understand the reactivity of Si–H bonds with alkyl radicals^10^ and the combustion in the presence of silane.

Due to the importance of the reaction (R2), and the lack of reliable experimental data, the main purpose of this work is to compute theoretical accurate values of the barrier heights, reaction energies, rate constants, and KIEs of the reactions (R2 to R2c):
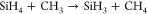
R2
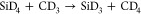
R2a
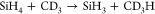
R2b
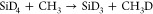
R2cIn this study, a dual-level strategy was employed to construct the minimal energy path (MEP). The kinetic rate constants were evaluated using the variational transition state theory with multidimensional tunneling corrections, which included microcanonically optimized multidimensional tunneling^19^ (μOMT).

The accuracy of the potential energy surface and the VTST method employed in the rate constants of the SiH_4_ + CH_3_ reaction was validated comparing the results calculated for the simpler reaction SiH_4_ + H, with those already validated in previous investigations.^2,5,9−13^

## Methods

The equilibrium geometry of stationary states (reactants, products, and transition states) of the system reactions from (R1) and (R2) were optimized using Dunning’s correlation consistent basis set with diffuse functions aug-cc-pVTZ^20,21^ employing the ωB97X-D and CCSD(T) methods.

The multireference character of all species was verified by using the *T*_1_ diagnostic calculated with the geometries optimized at the CCSD(T)/aug-cc-pVTZ level. According to Lee et al.,^22^*T*_1_ values greater than 0.02 and 0.045 for closed-shell and open-shell systems, respectively, indicate that the single-reference-based electron correlation methods are probably unreliable. The vibrational harmonic frequencies and zero-point energies (ZPE) were determined for all stationary states of the SiH_4_ + H and SiH_4_ + CH_3_ reactive systems using the ωB97X-D/aug-cc-pVTZ and CCSD(T)/aug-cc-pVTZ levels of theory.

To improve the accuracy of the energetic properties, using the geometries optimized at the CCSD(T)/aug-cc-pVTZ level, single-point calculations were carried out with the CCSD(T)/aug-cc-pVnZ (*n* = Q and 5) approach. For the silicon atom, the aug-cc-pV(n+d)Z (*n* = Q and 5)^21^ basis set was used. The energies were extrapolated using the complete basis set (CBS) extrapolation scheme method proposed by Halkier et al.^23^ as follows:
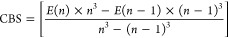
1where *E*(*n*) is the energy calculated with the aug-cc-pVnZ basis set. All the electronic calculations were carried out using the Gaussian 09 (G09)^24^ code.

Calculations of accurate energetic properties are essential to build the MEP, and they are defined as the classical barrier height (, electronic energy difference between the saddle point and reactants), vibrationally adiabatic barrier height () electronic energy difference between saddle point and reactants with zero-point energy corrections), the electronic energy of reaction (Δ*E* electronic energy difference between products and reactants), and the enthalpy of the reaction at 0 *K* ().

Thermal rate constants of all studied reactions were calculated employing the VTST with interpolated optimized corrections (VTST-IOC)^25^ approach. This procedure is called the dual-level method for which a low-level reaction energy path is interpolated by high-level electronic and vibrational frequencies.^26^ The low-level path was computed by the ωB97X-D/aug-cc-pVTZ methodology. The high-level electronic computations were carried out with the CCSD(T)/CBS_Q-5_ methodology, and the frequencies were computed with the CCSD(T)/aug-cc-pVTZ methodology.

The MEP is defined as the steepest descent path through the reaction coordinates (*s*, distance along the MEP) from the saddle point (*s* = 0) to the reactants (*s* < 0) and to the products (*s* > 0). The MEP for the reactive systems (R1) to (R2) have been calculated from *s* = −1.5 Å (reactant side) to *s* = +1.5 Å (product side). The reactional paths were computed within the curvilinear coordinate approach using Page and McIver’s steepest-descent stabilization method.^27^ The RODS algorithm was employed to reorient the generalized transition-state theory dividing surface with a step size of 0.02 Å and scale mass (μ) equal to 1 amu. The Hessians were calculated every 5 steps. The vibrationally adiabatic () potentials were determined as follows:
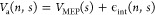
2where  is the potential energy of the MEP (with the zero at the reactant side) and  is the vibrational energy at *s* from the generalized normal-mode vibrations orthogonal to the reaction coordinate.

The VTST locates the dividing surface along the reaction coordinate (*s*) at the maximum of the energy of activation (), and the rate constants could be given by
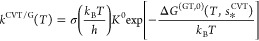
3where σ is the reaction-path symmetry number,^28^*h* and *k*_B_ are, respectively, the Planck and Boltzmann constants, and *K*^0^ is the reciprocal of the standard-state concentration expressed in molecule.cm^–3^. Values of σ are 4 and 12 for the (R1 and R2) paths, respectively.

It is well-known that tunneling through a barrier of potential contributes significantly to many chemical reaction processes at temperatures below 1000 K, especially for reactions involving a transfer of a hydrogen atom.^29^ To consider all these effects along the MEP, the rate constant  is multiplied by a ground-state transmission constant, , and the variational rate constant with quantum effects is given by
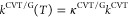
4In the present work, three semiclassical multidimensional tunneling methods were employed: the centrifugal dominant small-curvature semiclassical ground-state approximation^30^ (SCT), the large-curvature ground-state approximation^31^ (LCT), and the microcanonical optimized multidimensional tunnelling approximation^19^ (μOMT). In the μOMT approach, both small- and large-curvature tunneling are calculated, and the larger of the two approximations is used at each tunneling energy. The rate constants for reactions (R1) and (R2) computed by the CVT/μOMT method were fit with a model proposed by Truhlar and Zheng,^32^ given by
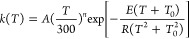
5which contains four parameters, *A*, *n*, *T*_0_, and *E*, where *A* is the pre-exponential constants, *E* (kcal.mol^–1^) is an energetic parameter, *T*_0_ (*K*) is the temperature,  is the pre-exponential temperature-dependent factor, and *R* is the gas constant equal to 1.987 × 10^–3^ kcal.mol^–1^K^–1^. The Arrhenius activation energy *E*_a_ can be computed by
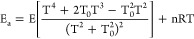
6Thermal rate constants were carried out with POLYRATE code version 17-C^33^ and GAUSSRATE interface^34^ for the potential energy surface calculations.

## Results and Discussion

### SiH_4_ + H SiH_3_ + H_2_

#### Structures and Energetics

In order to validate the accuracy of the methodology employed in this study, we first consider the reaction SiH_4_ + H SiH_3_ + H_2_, a well-studied prototype hydrogen abstraction reaction. The values of the optimized structural parameters (bond lengths, angles, and frequencies calculated with CCSD(T)/aug-cc-pVTZ) and the *T*_*1*_ diagnostic are listed in Table S3, Cartesian’s coordinates are provided in Table S2, and the harmonic frequencies of reactants and transition states, computed with ωB97X-D/aug-cc-pVTZ and CCSD(T)/aug-cc-pVTZ, for both reactions, are presented in Table S4 .

The geometrical parameters of all stationary states calculated with CCSD(T)/aug-cc-pVTZ, listed in Table S3, are in good agreement with the experimental data^35−37^ and theoretical values obtained by Cao et al.^12^ Moreover, all the bond lengths differ less than 0.04 Å, and the angles differ less than 0.8 degree. The vibrational imaginary frequency of the transition state computed with CCSD(T)/aug-cc-pVTZ (i1248 cm^–1^) is also in excellent agreement with the values employed for the potential surface of Cao et al.^12^ (i1237 cm^–1^) and bigger than the value computed by Fang et al.^18^ (i1123 cm^–1^) using the M06-2X/cc-pVTZ level. The values of the *T*_1_ diagnostic calculated with CCSD/aug-cc-pVTZ for all stationary species are less than 0.017, indicating the monoconfigurational character according to the recommendations of Lee et al.^22^

Table 1 lists the energetic data for the reaction (R1). Calculations with CCSD(T)/CBS_Q-5_ predict the vibrationally adiabatic barrier height (), enthalpy of formation at 0 K (), classical barrier height (), and the electronic energy of reaction (Δ*E*) to be equal to 4.4, – 13.0, 5.2, and −13.0 kcal/mol, respectively. The values of  and , calculated with CCSD(T)/CBS_Q-5_, agree with the experimental values of Goumri et al.,^9^ equal to 4.1 ± 0.7 and −12.9 ± 0.6 kcal/mol, respectively. The values of  and Δ*E*, calculated with CCSD(T)/CBS_Q-5_, agree with those obtained by Espinosa-García et al.^5^ (5.1 and −13.0 kcal/mol, respectively) and Fang et al.^18^ (4.9 and −13.4 kcal/mol, respectively) in previous theoretical calculations. These values differ by less than 1.0 kcal/mol from those of the CCSD(T)/CBS_Q-5_ calculations.

**Table 1:** Energetics (in kcal/mol) of the SiH_4_ + H Reaction Calculated from Different Methods and Basis Sets

Methods			Δ*E*	
ωB97X-D/aug-cc-pVTZ	5.0	4.7	–12.0	–11.8
CCSD(T)/aug-cc-pVTZ	5.0	4.2	–13.6	–13.6
CCSD(T)/aug-cc-pVQZ//CCSD(T)/aug-cc-pVTZ	5.2	4.4	–13.0	–13.0
CCSD(T)/aug-cc-pV5Z//CCSD(T)/aug-cc-pVTZ	5.2	4.4	–13.0	–13.0
CCSD(T)/CBS_Q-5_	5.2	4.4	–13.0	–13.0
Espinosa-García^5^	5.1	4.4	–13.1	–13.0
DLPNO-CCSD(T)/cc-pVTZ//M06-2X/cc-pVTZ^18^	···	4.9	–13.4	···
Experimental^9^	···	4.1± 0.7	···	-12.9±0.6
Hess’s Law^38−40^	···	···	···	–12.94

#### Rate Constants

The potential energy surface (*V*_MEP_) computed with the dual-level strategy has the max(*V*_MEP_) value equal to 5.2 kcal/mol which is in good agreement with that previously reported by Cao et al.^12^ (equal to 5.3 kcal/mol) and by Espinosa-García et al.^5^ (equal to 5.1 kcal/mol).

The thermal rate constants were computed at temperatures in the range from 200 to 1600 K and are presented in Table S5. These results can be compared with the previous theoretical values (at 300 K) computed by Cao et al.,^12^ Wang et al.,^11^ and Espinosa-García et al.^5^ which are equal to 1.72 × 10^–13^, 2.74 × 10^–13^, and 2.09 × 10^–13^ cm^3^molecule^–1^s^–1^, respectively. Experimental data measured at 300 K by Goumri et al.^9^ and Arthur et al.^13^ are equal to 2.81 × 10^–13^ and 3.46 × 10^–13^ cm^3^molecule^–1^s^–1^, respectively. At the same temperature, the present CVT/μOMT rate constant is equal to 2.13 × 10^–13^ cm^3^molecule^–1^s^–1^, which is in excellent agreement with the previous theoretical and experimental results.

**Figure 1:**
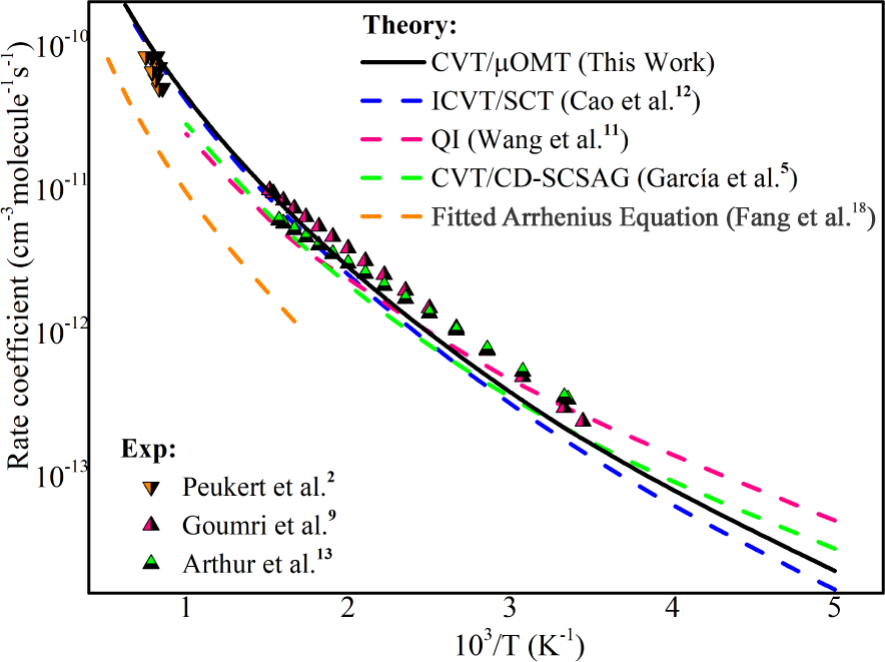
Arrhenius plot of the theoretical and experimental rate constant for SiH_4_ + H reaction at temperatures range of 200–1600 K.

Figure 1 gives the Arrhenius plot comparing the present CVT/μOMT rate constants with the previous theoretical^5,11,12,18^ and experimental data.^2,9,13^ At lower temperatures (in this work *T* < 400 K), as expected, the contributions of tunneling effects are important, due to the transfer of light hydrogen atom (H) along the MEP.

The comparable results of the rate constants computed using the ωB97X-D/aug-cc-pVTZ surface and previous ones^2,5,9−12,18^ reflect the good agreement involving barrier height, reaction energy, and the properties of the potential energy surface (PES), for instance, the imaginary frequency at the saddle point.

#### Kinetic Isotope Effects (KIEs)

The kinetic isotope effect is defined as the ratio of the rate constants corresponding, respectively, to the unlabeled and to the labeled species, that is, . A kinetic isotope effect, where  is larger than unity, is defined to be normal. It provides a sensitive test of several features of the shape of the MEP. In this work, for the reaction SiH_4_ + H, we have carried out the KIEs {/, /, /} presented in Tables S6–S8 with the numerical theoretical and experimental data values.

**Figure 2:**
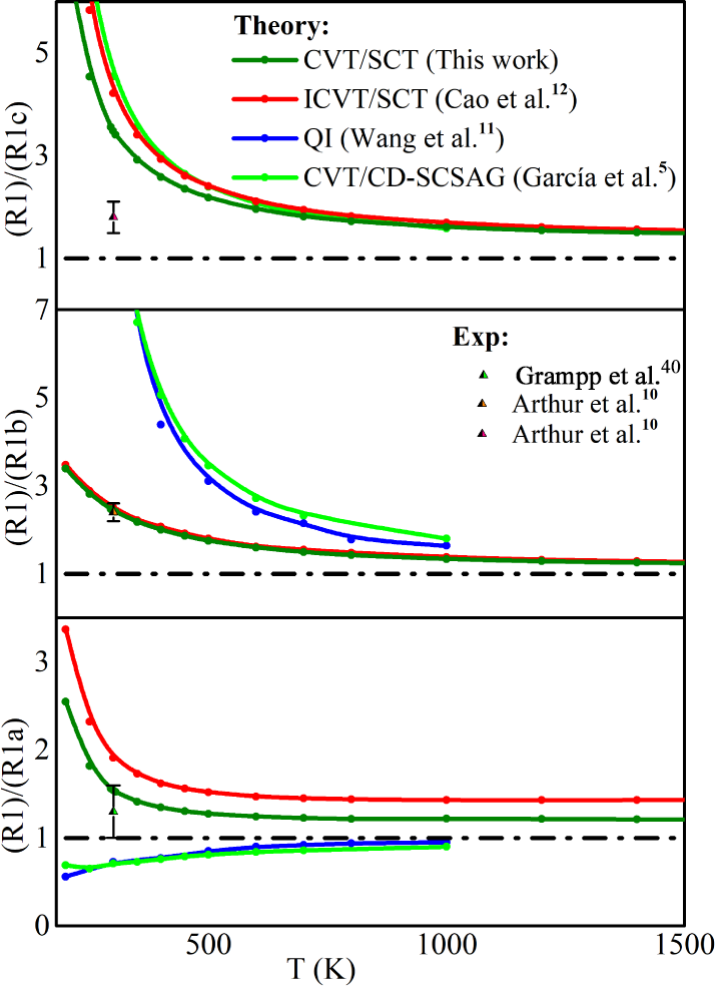
Kinetic isotope effects from the reaction SiH_4_ + H computed with the CVT/μOMT method (olive line) and previous theoretical and experimental data.

The KIEs from the reaction SiH_4_ + H are depicted in Figure 2. The thermal rate constant was calculated (R1) at the temperatures ranging from 200 to 1600 K provides the normal KIE (equal or larger than 1), which converges to closer to 1 at high temperatures as shown in Figure 2. All the ratio *k*_H_/*k*_D_ in this work is very similar to that one calculated by Cao et al.^12^

In our study, we observed a close agreement between the calculated values and the experimental data of KIEs. The estimated value of / was 1.54, demonstrating notable proximity to the experimentally measured value of 1.3 ± 0.3 by Grampp et al.^40^ Similarly, for /, we found a calculated value of 2.44, comparable to the experimental value of 2.4 ± 0.2 by Arthur et al.^10^ Additionally, for /, the estimated value was 3.48, while the experimental value was measured at 1.8 ± 0.3 by Arthur et al.^10^ This consistency between theoretical and experimental data reinforces our theoretical calculations’ validity and the proposed models’ applicability in describing the kinetic processes involved in the studied reactions. A discussion of the behavior of the KIE for these reactions has been included in Supporting Information.

### SiH_4_ +  SiH_3_ + CH_4_

#### Structures and Energetics

Values of the optimized structural parameters (bond lengths, angles, and frequencies) and values of the *T*_1_ diagnostic of the transition state are listed in Table 2. Cartesian’s coordinates are provided in Table S2. The structural parameters for TS_2_, calculated by CCSD(T)/aug-cc-pVTZ level, differed by less than 0.01 Å from the theoretical value computed by Fang et al.,^18^ computed by M06-2X/cc-pVTZ level. Values of the *T*_1_ diagnostic calculated with CCSD/aug-cc-pVTZ for all stationary species are less than 0.017, indicating the monoconfigurational character.

**Table 2:** Structural Properties (Bond Lengths in Å, Angles between Three Atoms in Degree, and Vibrational Frequencies in cm^–1^) of Reactants, Products, and Transition States (TS_2_) Calculated with CCSD(T)/aug-cc-pVTZ Level of Theory

Species	*T*_1_	Parameters	CCSD(T)	M06-2X^18^
TS_2_	0.016	R(Si–H)	1.486	1.480
		(HSiH)	109.4	109.4
		R(C–H)	1.085	1.081
		(HCH)	116.0	116.2
		R(Si–H*)	1.625	1.603
		R(C–H*)	1.560	1.595
		(SiH*C)	180.0	180.0

Species	Method	Frequencies
	CCSD(T)/aug-cc-pVTZ	i1292, 12, 185, 439, 519, 865, 945, 1060, 1070, 1435, 2219, 2236, 3076, 3231
	M06-2X/cc-pVTZ^18^	i1295, 22, 183, 459, 508, 870, 950, 1032, 1044, 1424, 2262, 2278, 3105, 3262

The energetic parameters for reaction (R2) are listed in Table 3, and our best values are calculated with the single-point CCSD(T)/CBS_Q-5_//CCSD(T)/aug-cc-pVTZ approach, which predicts classical barrier height () and energy of reaction (Δ*E*) equal to 9.0 and −15.8 kcal/mol, respectively. When we compare the formation enthalpy obtained using Hess’s law, we find that our calculations are within the chemical accuracy of approximately 0.5 kcal/mol. The theoretical results of Drozdova et al.,^41^ which employed the B3LYP/6-31++G** level, predict  and Δ*E* equal to 7.2 and −16.2 kcal/mol, respectively, and Fang et al.,^18^ using the DLPNO–CCSD(T)/cc-pVTZ//M06-2X/cc-pVTZ level, predict  and Δ*E* equal to 9.3 and −17.5 kcal/mol, respectively.

**Table 3:** Energetics (in kcal/mol) of the SiH_4_ + CH_3_ Reaction Calculated from Different Methods and Basis Sets

Methods			Δ*E*	
ωB97X-D/aug-cc-pVTZ	6.0	6.7	–16.7	–13.2
CCSD(T)/aug-cc-pVTZ	8.5	8.7	–16.4	–13.2
CCSD(T)/aug-cc-pVQZ//CCSD(T)/aug-cc-pVTZ	9.0	9.1	–15.7	–12.6
CCSD(T)/aug-cc-pV5Z//CCSD(T)/aug-cc-pVTZ	9.0	9.2	–15.8	–12.6
CCSD(T)/CBS_Q-5_//CCSD(T)/aug-cc-pVTZ	9.0	9.2	–15.8	–12.6
B3LYP/6-31++G**^41^^41^	6.7	7.2	–16.2	–12.8
DLPNO-CCSD(T)/cc-pVTZ//M06-2X/cc-pVTZ^18^	···	9.3	–17.5	···
Hess’s Law^39,42^	···	···	···	–13.0

#### Rate Constants

**Figure 3:**
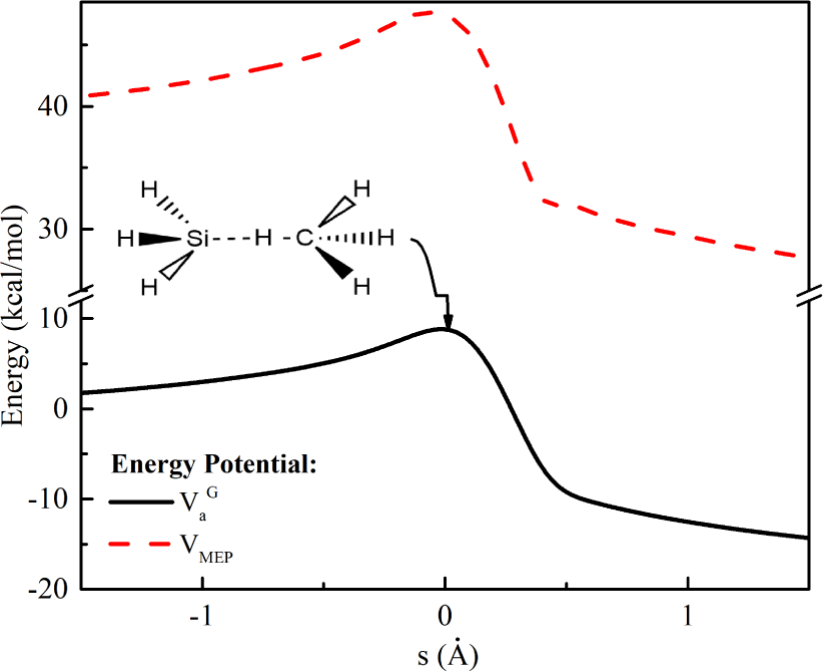
Potential energy surface (*V*_MEP_) and ground-state vibrationally adiabatic potential () from SiH_4_ + CH_3_ as a function of the reaction coordinate *s* computed with the dual-level strategy used in this work.

**Figure 4:**
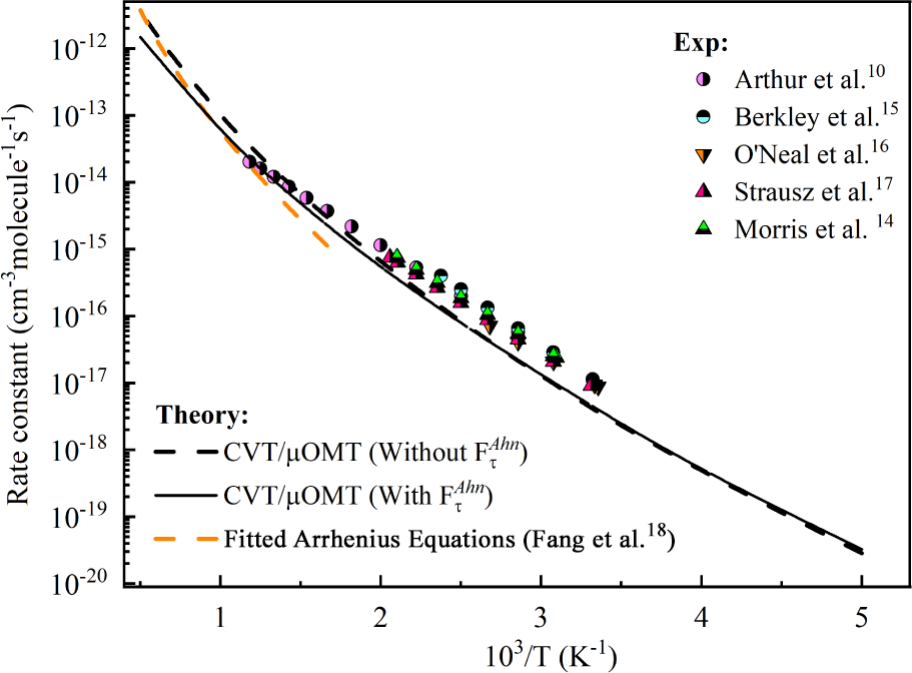
Arrhenius plot of the theoretical and experimental rate constant for SiH_4_ + CH_3_ reaction at the temperature range of 200–2000 K.

The classical (*V*_MEP_) and adiabatic () potential energy for the reaction (R2) computed with the dual-level strategy is presented in Figure 3, where the max (*V*_MEP_) equal to 9.0 kcal/mol is located at *s* = 0 Å. The CVT/μOMT thermal rate constants computed at the temperatures range of 200 to 2000 K are displayed in Table 4, together with experimental data. The Arrhenius plot comparing the present CVT/μOMT rate constants with the previous experimental data^10,14−17^ and theoretical values^18^ is presented in Figure 4.

**Table 4:** CVT/μOMT Rate Constants (in cm^3^molecule^–1^s^–1^) of the SiH_4_ + CH_3_ Path (R2), Including Contributions from Torsional Anharmonicity Factors () and Previous Results and Experimental Data (Power of 10 in Parentheses)^a^

	CVT/μOMT							
*T*(K)	Conventional		*k*(*T*)^b^^18^	Exp.^14^	Exp.^17^	Exp.^16^	Exp.^15^	Exp.^10^
200	2.8(−20)	3.3(−20)	···	···	···	···	···	···
250	4.6(−19)	4.9(−19)	···	···	···	···	···	···
300	4.0(−18)	3.8(−18)	···	···	···	9.0(−18)	···	···
350	2.1(−17)	1.9(−17)	···	5.4(−17)	4.5(−17)	4.1(−17)	6.5(−17)	5.7(−17)
400	8.3(−17)	7.6(−17)	···	1.9(−16)	1.6(−16)	···	2.0(−16)	2.0(−16)
450	2.6(−16)	2.0(−16)	···	4.9(−16)	4.2(−16)	···	···	5.3(−16)
500	6.6(−16)	5.3(−16)	···	···	···	···	···	1.1(−15)
600	3.0(−15)	2.2(−15)	1.2(−15)	···	···	···	···	3.7(−15)
700	9.6(−15)	8.8(−15)	4.1(−15)	···	···	···	···	8.6(−15)
800	2.4(−14)	1.6(−14)	1.2(−14)	···	···	···	···	1.6(−14)
900	5.3(−14)	3.2(−14)	2.7(−14)	···	···	···	···	···
1000	1.0(−13)	5.8(−13)	5.7(−14)	···	···	···	···	···
1500	9.4(−13)	4.5(−13)	7.4(−13)	···	···	···	···	···
2000	3.6(−12)	1.5(−12)	3.7(−12)	···	···	···	···	···

a The “conventional” column refers to the values without torsional anharmonicity factors.

b *k*(*T*) = (−5712.9/*RT*).

The transition state of TS2 is formed by two torsion modes, one about the forming H–Si bond and one about the C–H breaking bond. To treat this rotor, we adopt the single-torsional model^43^ for the independent torsion approximation, and the assumed torsion axis is represented by the Si–H–C bond. The torsional moment of inertia of this rotor (2.16 amu) was calculated using kpmoments, a utility of the MsTOR program.^44^ It is interesting to note that the two single CH_3_ and SiH_3_ rotors are connected only by weak van der Waals forces. The B97X-D and CCSD(T) calculations of the rotational barrier (*V*_barrier_) are lower than 30 cm^–1^ which characterize a free rotation (FR), i.e., . The quasi-harmonic VTST rate constants are corrected by the multiplicative factor () which is the ratio of anharmonic conformational-vibrational-rotational partition function () and quasiharmonic vibrational-rotational partition function (). As shown in Table 4 and Figure 3, this correction is significant at higher temperatures (0.450 at 1600 K), but it is very small at lower temperatures (1.054 at 300 K). Table S9 lists the values of  for all studied temperatures.

Compared with the experimental data, at temperatures in the range of 600 to 1600 K, the computed CVT/μOMT rate constants are in good agreement, as well as with the previous theoretical values of Fang et al.^18^ using a fitted Arrhenius equation. However, at temperatures in the range of 300 to 500 K, the difference between the set of experimental data increases. This discrepancy may be attributed to large tunneling effects, which may be underestimated by the computational methodologies. Another hypothesis for the source of the discrepancy is the method of evaluating the rate constants (R2) from the experimental data, which are deviated indirectly from the rate of radical recombination 2CH_3_ C_2_H_6_, resulting in considerable ascertains.

#### Kinetic Isotope Effects (KIEs)

**Figure 5:**
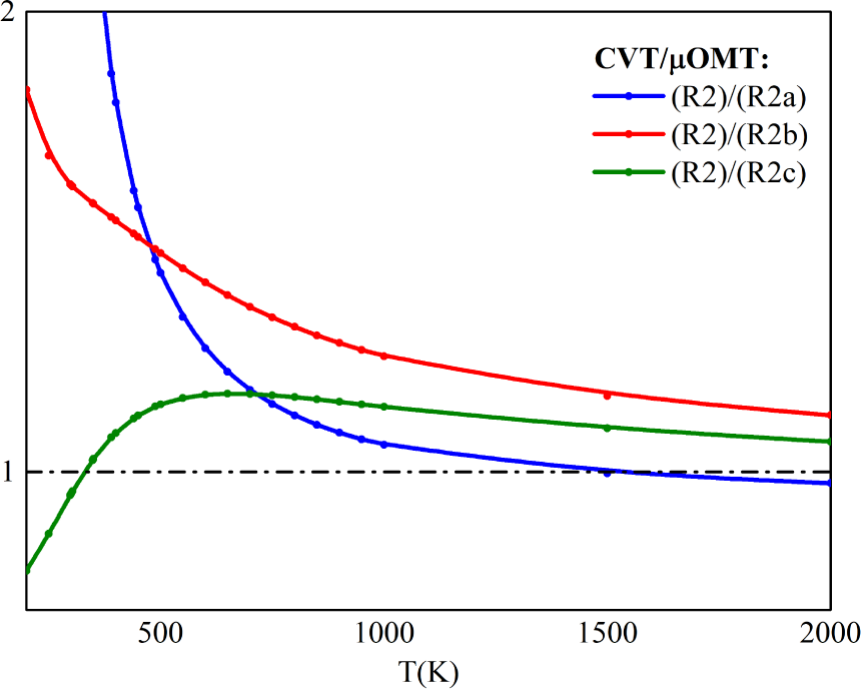
Kinetic isotope effects from the reaction SiH_4_ + CH_3_ computed with the CVT/μOMT method.

The rate constants obtained in this study are comparable to experimental measurements, validating our calculations of kinetic isotopic effects (KIEs) for reactions (R2a), (R2b), and (R2c). The thermal rate constant calculated for the response SiH_4_ + CH_3_ over the temperature range of 200 to 2000 K reveals that the normal KIEs {, , } converge to 1 at high temperatures. These results are detailed in Table S10 and illustrated in Figure 5.

#### The Fitted Kinetics Parameters for Reactions (R1) and (R2)

The CVT/μOMT rate constants from reactions were obtained by (R1) and (R2) and were used in order to fit a modified Arrhenius equation proposed by Truhlar and Zheng,^32^ given by eq 5. This equation was fitted in the temperatures range of 200–1600 K, and the resulting parameters are shown in Table 5. The root-mean-square residuals (RMSR) for both reactions (R1) and (R2) are less than 0.003.

**Table 5:** Fitted Kinetics Parameters Using eq 5 for Reactions (R1) and (R2)

	Reaction	
Parameter	R1	R2
A(s^–1^)	1.623 × 10^–16^	1.957 × 10^–21^
*n*	1.999	2.985
E(kcal.mol^–1^)	2.203	4.758
*T*_0_(K)	243.940	282.176
RMSR	0.0028	0.0012

## Conclusion

In this study, the DFT and CCSD(T) methods with various basis sets were employed to compute the electronic energies, equilibrium geometries, and vibrational frequencies of the stationary points of the H + SiH_4_ (R1) and CH_3_ + SiH_4_ (R2) hydrogen abstraction reactions. To improve the calculations of the electronic energies, an extrapolation to the complete basis was employed using the CCSD(T)/aug-cc-pVTZ optimized geometries and CCSD(T)/aug-cc-pV5Z single-point energies, as well as CCSD(T)/aug-cc-pV(5+d)Z single-point energies for silane compounds.

For reaction (R1), the evaluation of more accurate values of the classical barrier height, vibrationally adiabatic barrier, electronic energy of reaction, and enthalpy of formation at 0 K computed with CCSD(T)/CBS_Q-5_//CCSD(T)/aug-cc-pVTZ yielded values equal to 5.2, 4.4, −13.0, and −13.0 kcal/mol, respectively. These values agree well with the experimental values of vibrationally adiabatic barrier and enthalpy of formation at 0 K from Goumri et al.,^9^ which are equal to 4.1 ± 0.7 and −12.9 ± 0.6 kcal/mol, respectively. Furthermore, they are in exact agreement with the theoretical values calculated by Espinosa-García et al.,^5^ which are equal to 5.1, 4.4, −13.1, and −13.0 kcal/mol, respectively, and values of vibrationally adiabatic barrier and electronic energy of reaction computed by Fang et al.^18^ equal to 4.9 and −13.4 kcal/mol, respectively, which are in good agreement with the results of this study. For reaction (R2), our best estimated values for the classical barrier height, vibrationally adiabatic barrier, electronic energy of reaction, and enthalpy of formation at 0 K are equal to 9.0, 9.2, −15.8, and −12.6 kcal/mol, respectively. The barrier height and the vibrationally adiabatic barrier calculated in the present study differ by 2.0 kcal/mol from that previously calculated by Drozdova et al.,^41^ which was equal to 6.2 and 7.2 kcal/mol, respectively. This discrepancy is probably due to the different methodologies employed by them to optimize the transition state (B3LYP/6-31++G**). However, vibrationally adiabatic barrier computed by Fang et al.,^18^ equal to 9.3 kcal/mol, is in exact agreement with our previous calculations.

The CVT/μOMT rate constant for reaction (R1) is in excellent agreement with the previous experimental^2,9,10^ and theoretical^5,11,12^ results at temperatures in the range of 200–1600 K. Rate constants calculations for the reaction (R2) agree with the theoretical calculations by Fang et al.^18^ Experimental data^10,14−17^ are provided from indirectly results of the 2CH_3_ C_2_H_6_ recombination.^10^ Nevertheless, the experimental data are in good agreement with the present results, although there is a growing discrepancy at temperatures above 300 K.

The study of the KIEs shows that the ratios of the rate constants from reaction (R1) agree with the experimental values and are basically the same of the previous KIEs computed by Cao et al.^12^ which converge to 1 at high temperatures. The convergence of the KIEs for reactions (R1) and (R2) is shown. Based on the reliability of the same methodology employed to describe the kinetic properties of (R1), given the reliability of the methodology employed to describe the kinetic properties of (R1), it is reasonable to expect that the rate constants and KIEs for (R2) will also be highly accurate.

In particular, for reaction (R2), both the energetic parameters and the computed constants, including the determination of the parameters for the modified Arrhenius equation proposed by Truhlar and Zang,^32^ could prove useful for future theoretical and experimental work.

## Supporting Information Available

Figure S1: the potential energy surface  and ground-state vibrationally adiabatic potential () from SiH_4_ + H; Table S2: Cartesian coordinates (Å), electronic energies (*E*_e_) (a.u.), and zero-point correct energies (*E*_0_) of all species; Table S3: structural properties of reactants, products, and transition states calculated with CCSD(T)/aug-cc-pVTZ level of theory from SiH_4_ + H; Table S4: harmonic frequencies computed with the ωB97-XD and CCSD(T) of all species; Table S5: rate constants computed in this work of the SiH_4_ + H path and previous results and experimental data; Table S6: the kinetic isotope effects at different temperatures of the SiH_4_ + H path; Table S7: the kinetic isotope effects at different temperatures of the SiH_4_ + H path; Table S8: the kinetic isotope effects at different temperatures of the SiH_4_ + H path; Table S9: comparison of torsional anharmonicity factors at different temperatures; the torsional anharmonicity factor  is defined as the ratio of the anharmonic conformational-vibrational-rotational partition function  to the quasiharmonic vibrational-rotational partition function ; Table S10: the kinetic isotope effects at different temperatures of the SiH_4_ + CH_3_ path (PDF)
