# Supplementary material for: Hydrogen Atom Abstraction Reaction from Silane with Hydrogen and Methyl Radicals: Rate Constants and Kinetic Isotopic Effects
Source: J Phys Chem A. 2024 Nov 29;128(49):10489–97. doi: 10.1021/acs.jpca.4c05382 (PMC11647897; doi:10.1021/acs.jpca.4c05382)
Supplement: Supplementary file 1 — jp4c05382_si_001.pdf [file jp4c05382_si_001.pdf]

Hydrogen Atom Abstraction Reaction from Silane with Hydrogen and Methyl Radicals: Rate Constants and Kinetic Isotopic Effects

Filipe G. Kano,†,‡ Edson F. V. de Carvalho,¶ Luiz F. A. Ferrão,†,‡ Orlando Roberto-Neto,∗,§,‡ and Francisco B. C. Machado∗,†,‡

†*Departamento de Física, Instituto Tecnológico da Aeronáutica, 12228-900, José dos Campos, São Paulo, Brazil.*

‡*Laboratório de Computação Científica Avançada e Modelamento (Lab – CCAM), Instituto Tecnológico da Aeronáutica, 12228-900, José dos Campos, São Paulo, Brazil.*

¶*Departamento de Física, Universidade Federal do Maranhão, 65085-580, São Luís, Maranhão*

§*Divisão de Aerotermodinâmica e Hipersônica, Instituto de Estudos Avançados, São José dos Campos, 12228-001, São Paulo, Brazil.*

† *Laboratório de Computação Científica Avançada e Modelamento (Lab-CCAM), Instituto Tecnológico de Aeronáutica, 12228-900, São José dos Campos – SP, Brasil.*

E-mail: landoroberto@gmail.com; fmachado@ita.br

Support information

Supporting figures

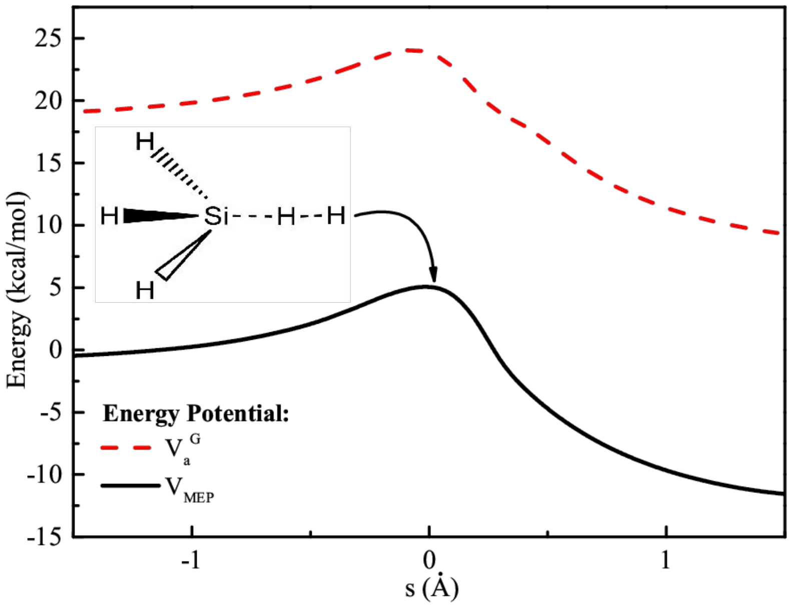

Figure S1: The potential energy surface (V_{MEP}) and ground-state vibrationally adiabatic potential (V_a^G) from SiH_4 + H· as function of the reaction coordinate *s* computed with the dual-level strategy used in this work.

Geometries and electronic energies of reactants and transition states (TSs)

Table S2: Cartesian coordinates (Å), electronic energies (E_e) (a.u.), and zero-point correct energies (E_0) of H, H_2, SiH_4, SiH_3, CH_4, CH_3 and transition states TS_1 (SiH_4+H) and TS_2 (SiH_4+CH_3) computed with the ωB97-XD and CCSD(T) methods and with the aug-cc-pVTZ basis set.

ωB97-XD/aug-cc-pVTZ.

H

E_e = -0.502803

H_2

Atomic Number	Coordinates (Angstroms)		
	X	Y	Z
1	0.000000	0.000000	0.371512
1	0.000000	0.000000	-0.371512

E_e = -1.1766497

E_0 = -1.166548

SiH_4

Atomic Number	Coordinates (Angstroms)		
	X	Y	Z
14	0.000000	0.000000	0.000000
1	0.856118	0.856118	0.856118
1	-0.856118	-0.856118	0.856118
1	-0.856118	0.856118	-0.856118
1	0.856118	-0.856118	-0.856118

E_e = -291.8894896

E_0 = -291.858706

SiH_3

Atomic Number	Coordinates (Angstroms)		
	X	Y	Z
14	0.000000	0.000000	0.080371
1	0.000000	1.412543	-0.375064
1	1.223298	-0.706271	-0.375064
1	-1.223298	-0.706271	-0.375064

E_e = -291.2347875

E_0 = -291.213728

CH_4

Atomic Number	Coordinates (Angstroms)		
	X	Y	Z
6	0.000000	0.000000	0.000000
1	0.628048	0.628048	0.628048
1	-0.628048	-0.628048	0.628048
1	-0.628048	0.628048	-0.628048
1	0.628048	-0.628048	-0.628048

E_e = -40.5201657

E_0 = -40.475156

CH_3

Atomic Number	Coordinates (Angstroms)		
	X	Y	Z
6	0.000000	0.000000	0.000000
1	0.000000	1.078365	0.000000
1	0.933892	-0.539183	0.000000
1	-0.933892	-0.539183	0.000000

E_e = -39.8391597

E_0 = -39.809373

TS_1 (SiH_4+H)

Atomic Number	Coordinates (Angstroms)		
	X	Y	Z
14	0.002816	0.005102	0.001959
1	0.004876	0.008836	1.483778
1	1.392871	0.008836	-0.511402
1	-0.735118	1.183118	-0.511402
1	-0.709817	-1.286303	-0.493801
1	-1.299913	-2.355652	-0.904316

E_e = -292.3842132

E_0 = -292.353963

TS_2 (SiH_4+CH_3)

Atomic Number	Coordinates (Angstroms)		
	X	Y	Z
6	0.000000	0.000000	-2.103989
1	-0.918026	0.530023	-2.322262
1	0.000000	-1.060045	-2.322262
1	0.918026	0.530023	-2.322262
1	0.000000	0.000000	-0.519746
14	0.000000	0.000000	1.094646
1	0.000000	1.397998	1.595142
1	1.210702	-0.698999	1.595142
1	-1.210702	-0.698999	1.595142

E_e = -331.7189478

E_0 = -331.657493

CCSD(T)/aug-cc-pVTZ.

H

E_e = -0.4998212

H_2

Atomic Number	Coordinates (Angstroms)		
	X	Y	Z
1	0.000000	0.000000	0.371512
1	0.000000	0.000000	-0.371512

E_e = -1.1726356

E_0 = -1.162609

SiH_4

Atomic Number	Coordinates (Angstroms)		
	X	Y	Z
14	0.000000	0.000000	0.000000
1	0.856118	0.856118	0.856118
1	-0.856118	-0.856118	0.856118
1	-0.856118	0.856118	-0.856118
1	0.856118	-0.856118	-0.856118

E_e = -291.4400685

E_0 = -291.408743

SiH_3

Atomic Number	Coordinates (Angstroms)		
	X	Y	Z
14	0.000000	0.079230	0.000000
1	-1.414926	-0.369741	0.000000
1	0.707463	-0.369741	-1.225362
1	0.707463	-0.369741	1.225362

E_e = -290.7888953

E_0 = -290.767618

CH_4

Atomic Number	Coordinates (Angstroms)		
	X	Y	Z
6	0.000000	0.000001	0.000000
1	-0.055897	0.807306	-0.730132
1	0.924177	-0.558952	-0.146432
1	-0.015939	0.418473	1.006274
1	-0.852340	-0.666831	-0.129709

E_e = -40.4409302

E_0 = -40.396129

CH_3

Atomic Number	Coordinates (Angstroms)		
	X	Y	Z
6	0.000000	0.000000	-0.000001
1	-0.137639	1.070688	0.000002
1	-0.858434	-0.654543	0.000002
1	0.996072	-0.416148	0.000002

E_e = -39.7636609

E_0 = -39.733955

TS_1 (SiH_4+H)

Atomic Number	Coordinates (Angstroms)		
	X	Y	Z
14	-0.154476	0.000006	0.000001
1	-0.627058	-0.801419	-1.155261
1	-0.627678	-0.599924	1.271392
1	-0.627919	1.400915	-0.116431
1	1.438689	0.000285	0.000218
1	2.606636	0.000065	0.000072

E_e = -291.9318788

E_0 = -291.901912

TS_2 (SiH_4+CH_3)

Atomic Number	Coordinates (Angstroms)		
	X	Y	Z
6	2.094448	-0.000009	0.000005
1	2.313374	0.567464	-0.898108
1	2.313441	-1.061493	-0.042497
1	2.313658	0.494057	0.940428
1	0.534267	0.000032	0.000123
14	-1.091244	0.000004	0.000009
1	-1.588033	1.400444	-0.029622
1	-1.587988	-0.674662	1.227576
1	-1.587985	-0.725836	-1.198055

E_e = -331.1902056

E_0 = -331.128894

Table S3: Structural properties (bond lengths in Å, angles between three atoms in degree and vibrational frequencies in cm^{-1}) of reactants, products, and transition state (TS_1) calculated with CCSD(T)/aug-cc-pVTZ level of theory.

Species	T_1	Parameters	X	Y[1]	Surface [1]	Expt.
SiH_4	0.011	R(Si−H)	1.483	1.480	1.480	1.481[2]
		∠(HSiH)	109.5			109.5[2]
SiH_3	0.015	R(Si−H)	1.484	1.481	1.481	1.468[3]
		∠(HSiH)	111.3	111.3	111.2	110.5[3]
H_2	0.005	R(H−H)	0.743	0.742	0.742	0.741[4]
TS_1	0.016	R(Si−H)	1.483	1.480	1.480	
		∠(HSiH)	110.3	110.3	110.3	
		R(Si−H^1)	1.593	1.594	1.594	
		R(H1-H2)	1.116	1.157	1.157	
		∠(SiH^1H^2)	180.0	180.0	179.9	

Species		Frequencies
$SiH_{4(T_d)}$	X	925, 980, 2249, 2255
	Y[1]	930, 983, 2261, 2265
	Surface[1]	926, 980, 2251, 2258
$SiH_{3(C_{3v})}$	X	764, 934, 2212, 2248
	Y[1]	769, 941, 2230, 2262
	Surface[1]	773, 942, 2220, 2259
$H_{2(D_{*H})}$	X	4401
	Y[1]	4403
	Surface[1]	4418
$TS_{1(C_{3V})}$	X	i1248, 278, 876, 935, 971, 1165, 2236, 2254
	Y[1]	i1277, 275, 882, 941, 977, 1162, 2250, 2265
	Surface[1]	i1237, 280, 882, 943, 980, 1159, 2248, 2262

X=CCSD(T)/aug-cc-pVTZ
Y=UCCSD(T)/cc-pVQZ

Frequencies of reactants and transition states (TSs)

Table S4: Harmonic frequencies (cm^{-1}) of H, H_2, SiH_4, SiH_3, CH_4, CH_3 and transition states TS_1 and TS_2 computed with the ωB97-XD and CCSD(T) methods and with the aug-cc-pVTZ basis set.

ωB97-XD/aug-cc-pVTZ.

Frequencies (in cm^{-1}) calculated with the ωB97XD /aug-cc-pVTZ method.

Species	Symmetry	Frequencies
SiH_4	T_d	917 917 917 981 981 2197 2200 2200 2200
SiH_3	C_{3v}	760 935 935 2182 2216 2216
H_2	D_{*H}	4434
CH_3	D_{3h}	506 1414 1414 3126 3307 3307
CH_4	T_d	1360 1360 1360 1577 1577 3042 3160 3160 3160
TS_1	C_{3v}	i 905 263 263 883 925 925 968 968 1396 2219 2233 2233
TS_2	C_{3v}	i1155 71 193 194 470 520 520 865 945 945 1057 1057 1066 1430 1430 2194 2208 2208 3096 3253 3253

CCSD(T)/aug-cc-pVTZ.

Frequencies (in cm^{-1}) calculated with the CCSD(T) /aug-cc-pVTZ method.

Species	Symmetry	Frequencies
SiH_4	T_d	925 925 925 980 980 2249 2255 2255 2255
SiH_3	C_{3v}	764 934 934 2212 2248 2248
H_2	D_{*H}	4401
CH_3	D_{3h}	496 1418 1419 3115 3295 3295
CH_4	T_d	1350 1350 1351 1573 1574 3028 3146 3147 3147
HD	D_{*H}	3812
D_2	D_{*H}	3113
SiD_4	T_d	680 680 680 693 693 1591 1630 1630 1630
SiD_3	C_{3v}	564 672 672 1571 1627 1627
CD_3	D_{3h}	2456 2456 2204 1044 1044 385
CD_3H	T_d	3121 2329 2329 2145 1325 1324 1059 1059 1029
CD_4	T_d	2329 2329 2329 2142 1113 1113 1021 1021 121
CH_3D	T_d	3146 3146 3062 2278 1512 1511 1345 1192 1192

$SiH_4D(TS_{R1a})$	C_{3v}	i1067 247 247 876 932 932 966 966 979 2236 2254 2254
$SiD_4H(TS_{R1b})$	C_{3v}	i1042 234 234 642 680 680 700 700 1004 1590 1630 1630
$SiD_5(TS_{R1c})$	C_{3v}	i883 199 199 641 680 680 689 689 852 1590 1630 1630
$SiD_4CD_3(TS_{R2a})$	C_{3v}	i976 8 132 132 380 381 381 638 680 680 767 767 826 1050 1050 1578 1616 1616 2183 2403 2403
$SiH_4CD_3(TS_{R2b})$	C_{3v}	i1281 14 169 169 391 418 418 858 880 944 944 1046 1046 1052 1053 2186 2219 2236 2236 2403 2404
$SiD_4CH_3(TS_{R2c})$	C_{3v}	i982 3 140 140 422 475 475 641 680 680 808 808 1016 1434 1435 1578 1616 1616 3076 3231 3231
TS_1	C_{3v}	i1248 278 278 876 935 935 971 971 1165 2236 2254 2254
TS_2	C_{3v}	i1292 12 185 185 439 519 519 865 945 945 1060 1070 1070 1435 1435 2219 2236 2236 3076 3231 3231

Rate constants of the SiH_4 + H

Table S5: CVT/μOMT rate constants (in $cm^3 molecule^{-1} s^{-1}$) of the $SiH_4 + H \rightarrow SiH_3 + H_2$ path and previous results and experimental data (power of 10 in parentheses).

T(K)	CVT/μOMT	k(T)*[5]	ICVT/SCT[1]	QI[6]	CVT/SCSAG[7]	Exp.[8]	Exp.[9]	Exp.[10]
200	2.1(-14)	···	1.6(-14)	4.8(-14)	3.0(-14)	···	···	···
250	7.7(-14)	···	6.0(-14)	···	8.8(-14)	···	···	···
298	2.0(-13)	···	···	···	···	···	···	3.4(-13)
300	2.1(-13)	···	1.7(-13)	2.7(-13)	2.1(-13)	···	2.8(-13)	3,5(-13)
350	4.9(-13)	···	4.1(-13)	···	4.3(-13)	···	7.2(-13)	···
369	6.4(-13)	···	···	···	···	···	···	8.2(-13)
400	9.7(-13)	···	8.3(-13)	9.7(-13)	8.0(-13)	···	1.4(-12)	···
415	1.2(-12)	···	···	···	···	···	···	1.5(-12)
434	1.5(-12)	···	···	···	···	···	···	1.6(-12)
450	1.7(-12)	···	1.5(-12)	···	1.3(-12)	···	2.4(-12)	···
478	2.3(-12)	···	···	···	···	···	···	2.6(-12)
500	2.8(-12)	···	2.5(-12)	2.4(-12)	2.1(-12)	···	3.8(-12)	···
700	1.2(-11)	2.3(-12)	1.1(-11)	7.6(-12)	8.0(-12)	···	···	···
800	2.0(-11)	4.0(-12)	1.8(-11)	1.2(-11)	2.8(-11)	···	···	···
900	3.0(-11)	6.3(-12)	···	1,8(-11)	···	···	···	···
1000	4.4(-11)	9.3(-12)	4.0(-11)	2.4(-11)	···	···	···	···
1196	7.9(-11)	···	···	···	···	7.0(-11)	···	···
1200	8.0(-11)	1.8(-11)	7.4(-11)	···	···	···	···	···
1222	8.4(-11)	···	···	···	···	8.3(-11)	···	···
1229	8.6(-11)	···	···	···	···	5.8(-11)	···	···
1262	9.3(-11)	···	···	···	···	6.5(-11)	···	···
1400	1.3(-10)	3.0(-11)	1.2(-10)	···	···	···	···	···
1600	1.9(-10)	4.5(-11)	1.8(-10)	···	···	···	···	···

*$k(T) = 4.878 \times 10^{6} T^{2.224} \exp(-2791.7/RT)$

Kinetic isotope effects (KIEs)

SiH_4 + H

Table S6: The kinetic isotope effects ($k_{(R1)}/ k_{(R1a)}$) at different temperatures.

	$k_{(R1)}/ k_{(R1a)}$				
T(K)	CVT/μOMT	ICVT/SCT[1]	CVT/SCT[4]	QI[10]	Expt.[1]
200	2.55	3.37	0.69	0.56	
250	1.82	2.32	0.65		
295	1.56				
300	1.54	1.91	0.71	0.73	1.3±0.3
305	1.52				
350	1.41	1.73	0.73		
400	1.35	1.62	0.76	0.77	
450	1.30	1.56	0.79		
500	1.27	1.52	0.81	0.85	
600	1.24	1.47	0.84	0.90	
700	1.23	1.45	0.86	0.92	
800	1.22	1.44		0.94	
1000	1.22	1.43	0.90	0.95	
1200	1.21	1.43			
1400	1.21	1.43			
1600	1.21	1.44			

Table S7: The kinetic isotope effects ($k_{(R1)}/ k_{(R1b)}$) at different temperatures.

	$k_{(R1)}/ k_{(R1b)}$				
T(K)	CVT/μOMT	ICVT/SCT[3]	CVT/SCT[4]	QI[10]	Expt.[1]
200	3.40	3.48	33.60	30.10	
250	2.82	2.89	16.63		
295	2.47				
300	2.44	2.51	9.81	8.57	2.4±0.2
305	2.41				
350	2.18	2.25	6.72		
400	2.00	2.07	5.07	4.39	
450	1.86	1.92	4.08		
500	1.75	1.80	3.46	3.11	
600	1.59	1.65	2.72	2.41	
700	1.49	1.55	2.32	2.15	

800	1.42	1.48		1.78
1000	1.34	1.38	1.80	1.64
1200	1.29	1.32		
1400	1.26	1.29		
1600	1.23	1.26		

Table S8: The kinetic isotope effects ($k_{(R1)}/ k_{(R1c)}$) at different temperatures.

	$k_{(R1)}/ k_{(R1c)}$			
T(K)	CVT/μOMT	ICVT/SCT[3]	CVT/SCT[4]	Expt.[8]
200	7.10	10.0	10.85	
250	4.54	5.83	6.42	
295	3.55			
300	3.48	4.21	4.54	1.8±0.3
305	3.40			
350	2.92	3.40	3.57	
400	2.58	2.93	3.01	
450	2.35	2.61	2.64	
500	2.18	2.40	2.39	
600	1.95	2.11	2.06	
700	1.81	1.94	1.85	
800	1.72	1.82		
1000	1.60	1.69	1.58	
1200	1.54	1.61		
1400	1.50	1.56		
1600	1.48	1.53		

$SiH_4 + CH_3$

Table S9: Comparison of torsional anharmonicity factors at different temperatures. The torsional anharmonicity factor, F_{τ}^{Anh}, is defined as the ratio of the anharmonic conformational-vibrational-rotational partition function Q_{τ}^{FR} to the quasiharmonic vibrational-rotational partition function Q_{τ}^{QH}.

**T(K)**	Q_{τ}^{FR}	Q_{τ}^{QH}	F_{τ}^{Anh}
250	2.785941889	2.414598428	1.15
280	2.948363763	2.708208068	1.09
290	3.000551243	2.805995628	1.07
298.15	3.042422022	2.885666889	1.05
400	3.523968720	3.879950215	0.91
500	3.939916804	4.854741832	0.81
600	4.315962616	5.828825087	0.74
800	4.983644357	7.775926008	0.64
1000	5.571883779	9.722315237	0.57
1100	5.843841008	10.69536411	0.55
1200	6.103692867	11.66834819	0.52
1300	6.352924956	12.64128239	0.50
1400	6.592741795	13.61417742	0.48
1500	6.824136082	14.58704109	0.47
1600	7.047937439	15.55987928	0.45
1700	7.264847626	16.53269649	0.44
1800	7.475466535	17.50549621	0.43
1900	7.680311765	18.47828120	0.42
2000	7.879833608	19.45105368	0.40

As described by Wu et al.[11], the torsional anharmonicity factor, F_{τ}^{Anh}, is defined as the ratio of the anharmonic conformational-vibrational-rotational partition function (Q_{τ}^{FR}) to the quasiharmonic vibrational-rotational partition function of the torsional mode in the transition state (Q_{τ}^{QH})

$$F_{\tau}^{Anh} = \frac{Q_{\tau}^{FR}}{Q_{\tau}^{QH}}$$

where

$$Q_{\tau}^{FR}(T) = \sqrt{\frac{8\pi k_B T I_{\tau}}{\sigma_{\tau}^2 h^2}}$$

and

$$Q_{\tau}^{QH} = \frac{e^{-\frac{\beta\hbar\omega}{2}}}{1 - e^{\beta\hbar\omega}}.$$

where I_{τ} is the torsional moment of inertia, calculated using *kpmoments*, a utility in the MsTOR program[13], which was found to be 2.16 amu Å^2.

Table S10: The kinetic isotope effects at different temperatures of the SiH_4 + CH_3 path.

T(K)	$k_{(R2)}$/ $k_{(R2a}$	$k_{(R2)}$/ $k_{(R2b)}$	$k_{(R1)}$/ $k_{(R2c)}$
200	0.78	18.4	1.83
250	0.86	5.62	1.69
300	0.95	3.09	1.62
350	1.03	2.21	1.58
400	1.08	1.80	1.55
450	1.12	1.57	1.51
500	1.15	1.43	1.48
600	1.17	1.27	1.41
700	1.17	1.18	1.36
800	1.16	1.12	1.32
900	1.15	1.09	1.28
1000	1.14	1.06	1.25
1500	1.09	1.00	1.17
2000	1.07	0.98	1.12

Qualitative Discussion of the Kinetic isotope Effects (KIE)

Considering the rate constant as given by the Eyring equation with a tunneling coefficient correction:

$$k(T) = \kappa_{tun}\left(\frac{k_b T}{h}\right)\left(\frac{RT}{P^\circ}\right)e^{\frac{\Delta S^{\neq}}{R}}e^{\frac{-\Delta H_0^{\neq}}{RT}}$$

Calculating the ratio of the rate constants for the hydrogen and deuterated reactions at a given temperature, considering the molar entropy to be approximately the same:

$$KIE = \frac{k(T)_H}{k(T)_D} \sim \frac{\kappa_{tunH}}{\kappa_{tunD}} * \frac{e^{\frac{-\Delta H_{0\,H}^{\neq}}{RT}}}{e^{\frac{-\Delta H_{0\,D}^{\neq}}{RT}}}$$

$$KIE = \frac{k(T)_H}{k(T)_D} \sim \frac{\kappa_{tunH}}{\kappa_{tunD}} * e^{\frac{-(\Delta H_{0\,H}^{\neq} - \Delta H_{0\,D}^{\neq})}{RT}}$$

And considering the Wigner correction for the tunneling:

$$\kappa_{tun} = 1 + \frac{1}{24}\left(\frac{h\nu_i}{k_b T}\right)^2$$

Therefore:

$$\frac{\kappa_{tunH}}{\kappa_{tunD}} = \frac{1 + \frac{1}{24}\left(\frac{h\nu_{iH}}{k_b T}\right)^2}{1 + \frac{1}{24}\left(\frac{h\nu_{iD}}{k_b T}\right)^2}$$

Substituting into the KIE equation:

$$KIE \sim \frac{1 + \frac{1}{24}\left(\frac{h\nu_{iH}}{k_b T}\right)^2}{1 + \frac{1}{24}\left(\frac{h\nu_{iD}}{k_b T}\right)^2} * e^{\frac{-(\Delta H_{0\,H}^{\neq} - \Delta H_{0\,D}^{\neq})}{RT}} \qquad (1)$$

The estimates for the KIE presented in the next pages are based on the energetics obtained from CCSD(T)/aug-cc-pVTZ, as given in Table S11.

Table S11: Energetics (in kcal/mol) of the SiH_4 + deuterated radical reactions calculated with CCSD(T)/aug-cc-pVTZ. The non-deuterated reactions are also shown as reference. The imaginary frequency at the saddle point (v_i) is also included (in cm^{-1})

Label	System	v_i	$V^{\#}$	V_a^g	ΔE	ΔH_0
R1	SiH_4+H	1248	5.0	4.2	-13.6	-13.6
R1A	SiH_4+D	1067		3.8		-14.4
R1B	SiD_4+H	1042		4.8		-12.7
R1C	SiD_4+D	883		4.4		-13.7
R2	SiH_4+CH_3	1292	8.5	8.7	-16.4	-13.2
R2A	SiD_4+CD_3	976		8.7		-12.1
R2B	SiH_4+CD_3	1281		8.1		-13.9
R2C	SiD_4+CH_3	982		9.3		-17.1

Equation (1) estimates the KIE when not considering variational effects or multidimensional tunneling. Therefore, it represents what to expect when using a highly correlated potential energy surface to feed a simple kinetics methodology. We can also separate the influence of the variation in the imaginary frequency and the adiabatic barrier when using a deuterated system. The following plots are given for R1-2(A-C):

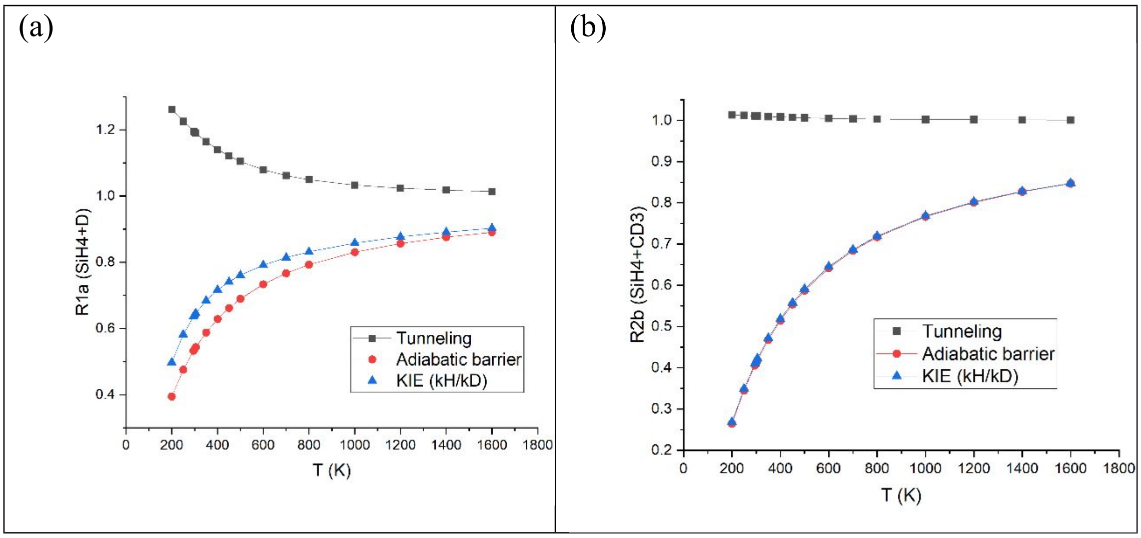

Figure S2. KIE effects for Silane + deuterated radical reaction systems. (a) SiH4+D (R1A). (b) SiH4+CD3 (R2B). Besides the total KIE (in blue), the figure also includes the contributions from the Tunneling (in black) and adiabatic barrier (in red).

From Figure S2, one can see that the contribution of the tunneling is low for R1A and minimal for R2B, and the KIE is dominated by the differences in the adiabatic barrier. This is expected since the imaginary frequency related to the breakage of the Si-H bond is not directly related to the deuterated atoms, especially in the case of the reaction with the DH3 radical. On the other hand, the ZPE at the saddle point is the lowest among the deuterated reactions, causing the largest lowering in adiabatic barriers, equal to -0.4 and -0.7 kcal/mol for R1A and R2B, respectively, when compared to the non-deuterated

reactions. This causes a considerable increase in the reaction rate of deuterated reactions, which in turn present an inverse KIE behavior. However, it is worth noting that more sophisticated tunneling approaches should significantly increase the tunneling of the non-deuterated reactions, especially for R1, overcoming the adiabatic effect and making the system present normal KIE behavior.

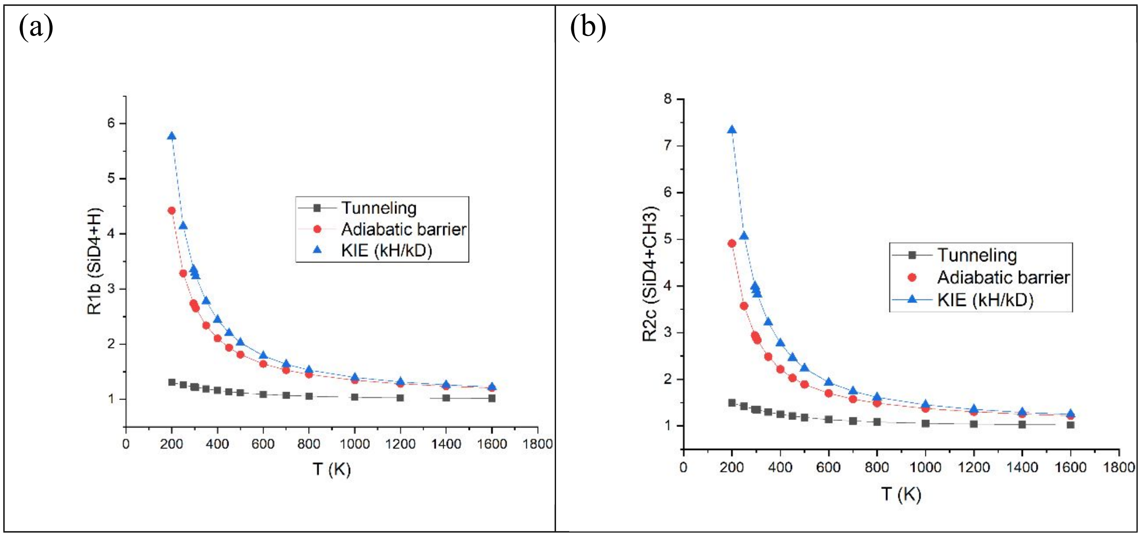

Figure S3. KIE effects for Deuterated silane + hydrogen radical reaction systems. (a) SiD4+H (R1B). (b) SiD4+CH3 (R2C). Besides the total KIE (in blue), the figure also includes the contributions from the Tunneling (in black) and adiabatic barrier (in red).

From Figure S3, one can see that the KIE for the deuterated silane + hydrogen radical is very high and has contributions from both tunneling and adiabatic barrier. However, the total KIE is still dominated by the latter. For these systems, the ZPE at the reactant (deuterated silane) is lower when compared to silane, causing a higher adiabatic barrier when reacting with the non-deuterated radical (H or CH_3), lowering the reaction rate. Since there is no competition between tunneling and adiabatic barrier effects, a normal KIE behavior is expected for all temperatures.

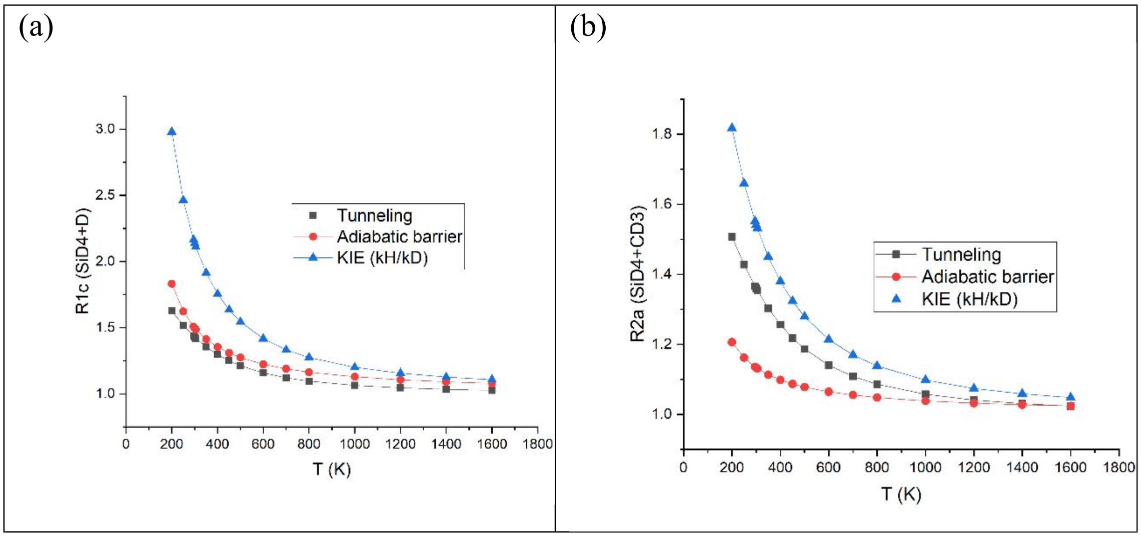

Figure S4. KIE effects for Deuterated silane + deuterated radical systems. (a) SiD4+D (R1C). (b) SiD4+CD3 (R2A). Besides the total KIE (in blue), the figure also includes the contributions from the Tunneling (in black) and adiabatic barrier (in red).

Concerning the all-deuterated reaction system shown in Fig S4, one can see that in this case there is no clear dominant contribution from the adiabatic barrier and perhaps the tunneling would be dominant if a more rigorous method was used. This is expected since, when all hydrogens are changed to deuterium, the ZPE of the reactants and saddle point are lowered more or less equally, causing little modification in the adiabatic barrier. As the tunneling is higher when the reaction involves only hydrogen, this contribution is significant and the total KIE depends on both terms, with total values expected to be lower than the previous reaction system, depending on the size of the rigorous tunneling effects.

We can also compare these estimates with previous theoretical and experimental data and the calculated KIE using CVT/μoMT.

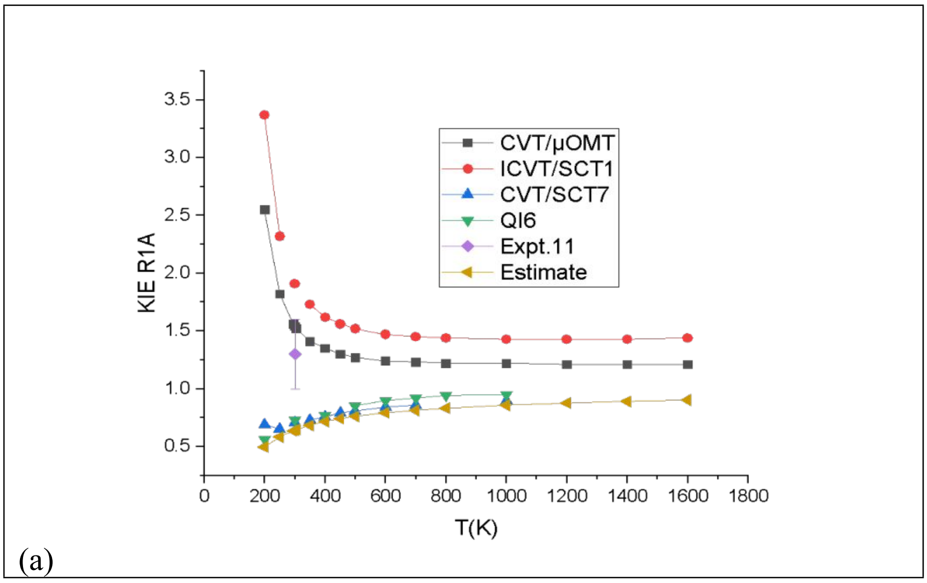

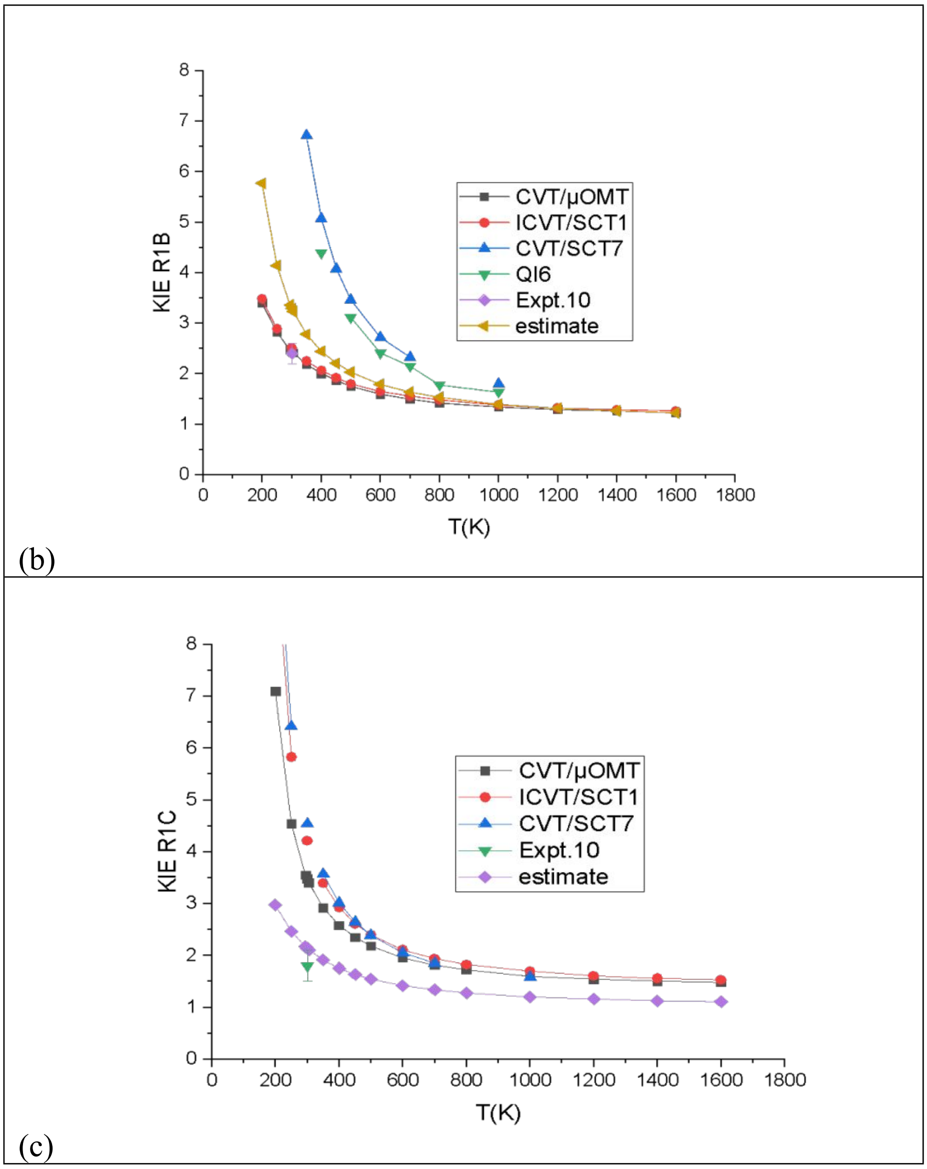

Figure S5. Comparison between KIE obtained with highly accurate method (CVT/µoMT) with previous theoretical and experimental data and estimates from energetic data of SiH4 + H reactions. (a) SiH4+D (R1A), b) SiD4+H (R1B), c) SiD4+D (R1C).

Overall, one can see that for R1 the estimates presented here, based on the energetics obtained from CCSD(T)/aug-cc-pVTZ and treated with a simplified TST/W kinetics, agree well with the previous theoretical data. However, this approach underestimates the tunneling effects, which is recovered only when using more accurate SCT or MT approaches. This is especially important for the R1A reactions, in which there is a competitive behavior between the tunneling and the adiabatic barrier in the KIE, and if the tunneling is not correctly accounted, the calculations would estimate an inverted KIE for this reaction, in disagreement with the (indirect) experimental data.

References

(1) Arthur, N. L.; Miles, L. A. Arrhenius parameters for the reaction of H atoms with SiH_4. *J. Chem. Soc.*, Faraday Trans. **1997**, 93, 4259-4264.

(2) Boyd, D. R. J. Infrared spectrum of Trideuterosilane and the Structure of the Silane Molecule. *The Journal of Chemical Physics* **1955**, 23, 922-926.

(3) Cao, J.;, Zhang, Z.; Zhang, C.; Bian, W.; Guo, Y. Kinetic study on the H + SiH_4 abstraction reaction using an ab initio potential energy surface. *The Journal of Chemical Physics*. **2011**, 134, 02315.

(4) Espinosa-García, J.; Sanson, J.; Corchado, J. C. The SiH_4+ H→ SiH_3+ H_2 reaction: Potential energy surface, rate constants, and kinetic isotope effects. *The Journal of Chemical Physics* **1998**, 109, 466-473.

(5) Fang, Q; Zhang, Y.; Xia, J.; Li, Y. Theoretical Investigation on H-abstraction Reactions of Silanes with H and CH3 Attacking: A Comparative Study with Alkane Counterparts. ACS Omega **2022**, 7, 5558-5569.

(6) Goumri, A.; Yuan, W.-J.; Ding, L.; Shi, Y.; Marshall, P. Experimental and theoretical studies of the reaction of atomic hydrogen with silane. *Chemical Physics* **1993**, 177, 233-241.

(7) Huber, K. P.; Herzberg, G. Molecular Spectra and Molecular Structure; Springer US, 1979.

(8) Mihelcic, D.; Schubert, V.; Schindler, R.; Potzinger, P. Rate constants for the reaction of hydrogen and deuterium atoms with silane. *The Journal of Physical Chemistry* **1977**, 81, 1543-1545, 1977.

(9) Peukert, S.; Herzler, J.; Fikri, M.; Schulz, C. High-Temperature Rate Constants for H+ Tetramethylsilane and H+ Silane and Implications about Structure–Activity Relationships for Silanes. *International Journal of Chemical Kinetics*, **2017**, 50, 57-72.

(10) Wang, W.; Feng, S.; Zhao, Y. Quantum instanton evaluation of the thermal rate constants and kinetic isotope effects for SiH4+ H→ SiH_3+ H_2 reaction in full Cartesian space. *The Journal of Chemical Physics* **2007**, 126, 114307.

(11) Wu, J.; Gao, L. G.; Ren, W.; Truhlar, D. G. Anharmonic kinetics of the cyclopentane reaction with hydroxyl radical. *Chemical Science*. **2020**, 11, 2511–2523.

(12) Yamada, C.; Hirota, E. Detection of the Silyl Radical SiH_3 by Infrared Diode-Laser Spectroscopy. *Physical Review Letters* **1986**, 56, 923-925.

(13) Zheng, J.; Mielke, S. L.; Clarkson, K. L.; Truhlar, D. G. MSTor: A program for calculating partition functions, free energies, enthalpies, entropies, and heat capacities of complex molecules including torsional anharmonicity. *Computer Physics Communications* **2012**, 183, 1803-1812.
